# Quantifying maternally derived respiratory syncytial virus specific neutralising antibodies in a birth cohort from coastal Kenya

**DOI:** 10.1016/j.vaccine.2015.02.039

**Published:** 2015-04-08

**Authors:** Joyce U. Nyiro, Charles Sande, Martin Mutunga, Patience K. Kiyuka, Patrick K. Munywoki, J. Anthony G. Scott, D James Nokes

**Affiliations:** a Kenya Medical Research Institute (KEMRI)—Wellcome Trust Research Programme, Centre for Geographic Medicine Research-Coast, Kilifi, Kenya; b London School of Hygiene and Tropical Medicine, London, UK; c School of Life Sciences, University of Warwick and WIDER, Coventry, UK

**Keywords:** Vaccine design, Respiratory syncytial virus, Maternal antibody, Birth cohort, Neutralisation, Rate of decay

## Abstract

**Background:**

Severe respiratory syncytial virus (RSV) disease occurs predominantly in children under 6 months of age. There is no licensed RSV vaccine. Protection of young infants could be achieved by a maternal vaccine to boost titres of passively transferred protective antibodies. Data on the level and kinetics of functional RSV-specific antibody at birth and over the early infant period would inform vaccine product design.

**Methods:**

From a birth cohort study (2002–2007) in Kilifi, Kenya, 100 participants were randomly selected for whom cord blood and 2 subsequent 3-monthly blood samples within the first year of life, were available. RSV antibodies against the A2 strain of RSV were assayed and recorded as the logarithm (base 2) plaque reduction neutralisation test (PRNT) titre. Analysis by linear regression accounted for within-person clustering.

**Results:**

The geometric mean neutralisation antibody titre was 10.6 (SD: 1.13) at birth with a log-linear decay over the first 6 months of life. The estimated rate of decay was −0.58 (SD: 0.20) log_2_PRNT titre per month and a half-life of 36 days. There was no significant interaction between cord titre and rate of decay with age. Mean cord titres rose and fell in a pattern temporally tracking community virus transmission.

**Conclusions:**

In this study population, RSV neutralising antibody titres decay approximately two-fold every one month. The rate of decay varies widely by individual but is not related to titre at birth. RSV specific cord titres vary seasonally, presumably due to maternal boosting.

## Highlights

• We report on the characteristics of RSV neutralising antibodies in a birth cohort.
• The levels of cord RSV antibodies increase during seasonal virus transmission.
• The rate of decay of RSV antibodies following birth is independent of cord titre.
• There is a high degree of variation between individuals in rate of decay.
• Important baseline data to maternal RSV vaccine development are provided.

## 1 Background

Severe RSV disease occurs primarily in infancy and predominantly among children 1–5 months of age [1]. Globally, RSV disease may be responsible for nearly 200,000 deaths per year in young children and 99% of these deaths occur in low income countries [2]. An annual rate of hospitalisation with RSV associated pneumonia of 1–2 per 100 for children in the first year of life has been reported in a rural community in Coastal Kenya [1,3].

Prevention of RSV disease is primarily targeted towards young infants [4], for which two vaccine strategies are appropriate: first, to vaccinate infants at the earliest age when able to develop a protective immune response with minimal reactogenicity, and second, to boost the level of RSV-specific antibodies in pregnant women before delivery to extend the duration of protective antibodies in early infancy [5–7].

There is evidence which supports the idea that maternal specific RSV antibodies provide protection from RSV disease [7,8]. Glezen et al. demonstrated that infants born with higher levels of antibody develop infection at a later age, and infants infected in the presence of moderate levels of serum antibody have milder illnesses than infants infected with lower or undetectable levels of antibody [7]. A case-control study in rural Mozambique showed that high levels of antibodies of maternal origin were associated with protection against RSV disease [5]. A randomised double blind placebo controlled trial among premature infants and infants with bronchopulmonary dysplasia showed that monthly prophylaxis with Palivizumab (a humanised monoclonal IgG antibody) was associated with a 55% reduction in hospitalisation as a result of RSV [9]. In addition, there are data that suggest a defined level of neutralising antibody which protects against RSV disease [10].

The success of a maternal RSV vaccination strategy will be governed by the degree to which a vaccine will boost levels of protective antibodies transferred by the mother to the infant and by the rate at which those antibodies decay. In this study we provide baseline data necessary to evaluate vaccine potential. Specifically, in a birth cohort of infants from Kilifi in Kenya, we estimate the mean of and variation in RSV neutralising (i.e. functional) antibody in newborn cord blood, and its rate of decay in relation to starting titre.

## 2 Methods

### 2.1 Study site and population

This study was conducted at the Kenya Medical Research Institute (KEMRI)—Wellcome Trust Collaborative Research Programme, in Kilifi, coastal Kenya [11]. Between 1999 and 2007, a birth cohort study [12,13] was conducted to investigate susceptibility to invasive pneumococcal disease among children aged 0 to 23 months. This study was a natural history study, with no intervention, in which cord and 3 monthly blood samples were collected for each participant.

The current study used archived serum samples of children who were participants of the Kilifi Birth Cohort (KBC) study and who were residents of the Kilifi Health and Demographic Surveillance System (KHDSS). Characteristics of this study population have been described before [11,12,14].

Approximately 6000 KBC study participants were recruited in the maternity ward and at the maternal and child health clinic at Kilifi District Hospital (KDH). A random sample of 100 participants from the KBC study, recruited from the KDH maternity ward, were selected from the study database regardless of RSV disease. Each selected participant had at least 3 blood samples (specifically, a cord blood and two follow up samples) each separated by approximately 3 months.

The temporal pattern of cases of RSV associated severe pneumonia was derived from continuous surveillance of children under 5 years of age admitted to Kilifi District Hospital, Kilifi, which serves the KHDSS population [1,15]. An RSV epidemic was empirically defined to begin when at least 10% of tested samples were RSV positive or with the observation of at least 2 cases of RSV infection per week in each of 2 consecutive weeks and were defined to continue as long as these conditions were satisfied, with the requirement that an epidemic must last for ≥4 weeks as previously defined [1]. These data were used to compare with the temporal pattern of cord titres.

The Kenya National Ethical Review Committee approved this study.

### 2.2 Laboratory procedures

Archived serum samples had been stored at −80 °C. The titre of RSV neutralising antibodies was determined by a plaque reduction neutralisation test (PRNT) as described previously [16]. The method incorporated a step in which serum samples were incubated at 56 °C in a water bath for 30 min to inactivate complement cascade proteins. The dilution of a test serum sample required to induce 50% neutralisation of a known titration of RSV A2 virus was determined using the Spearman Karber method [16].

### 2.3 Statistical analyses

Data analysis was conducted using STATA version 11.2 (College Station, Texas). The laboratory data were merged with archived data from the KBC and KHDSS databases for analysis. Sample PRNT titres were logarithmically transformed (base 2) for all statistical analyses. The titre of cord, first and second samples for an individual were defined as TC, T1 and T2, respectively. To offset bias on the rate of decay arising from RSV infection, we applied the following criteria. For individuals with T1 ≥ TC, all results for that individual were excluded, and for individuals with T2 ≥ T1 the result for sample T2 was excluded. In addition, all samples collected at ages ≥7 m after birth were excluded due to there being few in number and of wide age range (7–11 m) and a high likelihood that the measured antibody would be active rather than passive. The rate of decay of RSV specific log_2_PRNT titres from birth to <7 months of life was determined by simple linear regression, accounting for clustering of titres for samples from the same individual using the procedure for Huber–White sandwich estimator. Possible interaction between cord antibody titre and rate of decay of maternal antibodies was tested using the model with individuals categorised by quartile of titre at birth. Age dependence in the rate of decay of antibody titres was evaluated by comparing model fits using linear and quadratic terms for age. The Wald test was used to evaluate the significance of removal of variables in nested models. To evaluate the effect of infection on the antibody levels, the rate of decay of those participants with at least 2 samples collected within an RSV epidemic was estimated and compared to that of participants with at least 2 samples collected outside an RSV epidemic. A two sample *t*-test was used to compare the levels of neutralising antibodies and the mean of the rates of decay between samples collected within and outside an RSV epidemic and between binary covariates for birthweight (low birth weight <2.5 kg) measured using a weighing scale at birth and gestation period (premature < 37 weeks) based on date of last menstruation or by clinical evaluation.

## 3 Results

A total of 300 samples from 100 cohort participants were selected. For 8 individuals, the second follow-up sample (T2) was not available, therefore, 292 samples were screened for RSV specific neutralising antibodies. Applying the exclusion criteria as described in the Methods, 18 additional samples were removed (9 samples with T2 ≥ T1 and 9 samples ≥7 months), leaving 274 samples from 100 participants (76 with 3 samples, 22 with 2 samples and 2 with 1 sample) for analysis.

**Fig. 1:**
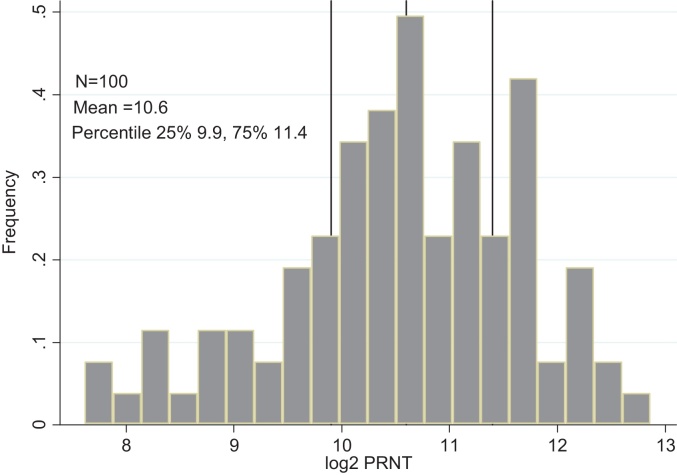
Frequency distribution of maternally transferred RSV specific antibodies (log_2_ transformed PRNT titres) at birth for 100 infants born in Kilifi, Kenya. The overall mean (variance) and upper and lower quartile titres (log_2_PRNT) are shown.

The frequency distribution of log-transformed PRNT titres for cord blood samples was approximately normal (Fig. 1) with mean concentration 10.6 (95% Confidence Interval (CI) 10.3–10.7, variance 1.28) and median of 10.6 (Interquartile Range (IQR) 9.95–11.4, 10th percentile 8.87 log_2_PRNT). The mean birth weight in kilograms of the participants in this study was 2.89 kg (SD: 0.49) and 19% were born with low birthweight <2.5 kg (mean: 2.16; SD: 0.21). Only 63 participants had data on gestational period. The mean gestation period in weeks was 38.6 (SD: 3.16). Of the 63 individuals with gestational data, 15(24%) were born prematurely i.e. <37weeks (mean 34.4; SD: 2.48). There was a significant difference in the cord blood neutralising antibody concentrations among the low birth weight, 9.9 log_2_PRNT compared to the normal weight 10.7log_2_PRNT (*P* = 0.02, *t* = −2.37) and between premature, 9.8 log_2_PRNT and not premature, 10.9 log_2_PRNT (*P* = 0.002, *t* = −3.18).

**Fig. 2:**
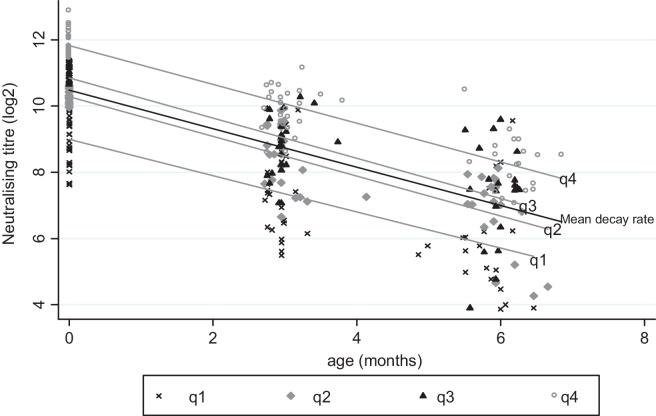
The decay in PRNT titre (log base 2 transformed) over the first 6 months of life for a birth cohort, Kilifi, Kenya, with best fit linear decay model for all samples (mean) and for each quartile cord level (q1–q4) assuming a fixed rate of decay (−0.58 log_2_PRNT titre per month).

The distribution of log-transformed PRNT titres by age is shown in Fig. 2, with the best fit linear regression model (age range 0–<7 m) giving an average reduction per month in log_2_PRNTof −0.58 (95%CI: −0.65 and −0.51), and a predicted mean titre at birth of 10.5 (95%CI 10.2 and 10.7). The estimated rate of decay for participants for whom all samples were collected within an RSV epidemic (*n* = 48; Samples = 98) was −0.56 log_2_PRNT per month (95%CI: −0.66 and −0.46) while those whose samples were all collected outside an RSV epidemic (*n* = 31; Samples = 66) was −0.67 log_2_PRNT per month (95%CI: −0.81 and −0.53). The difference in the rates of decay between those within an RSV epidemic and outside an epidemic was not statistically significant (*P* = 0.19, *t* = −1.32). The estimated rate of decay for samples collected in children under 4 m of age was −0.64 log_2_PRNT per month (95%CI: −0.70 and −0.58). We explored possible age-dependence in the rate of decay of antibody titre by fitting a quadratic term for age in the regression model. The estimate had a significant result (Wald test *P* = 0.001) and was not considered further, since the plots for the two models appeared similar with a predicted starting titre of 10.5 log_2_PRNT. The different rates of decay with their corresponding half life and mean duration are detailed in Table 1.

Results of regression analysis of the rate of decay of RSV antibodies by age and by quartile at birth (i.e. cord titre) is also shown in Fig. 2. There was no evidence that slope varied by quartile, i.e. no evidence of an interaction (Wald test comparing models with age and cord titre with or without interaction, *P* = 0.70.).

**Fig. 3:**
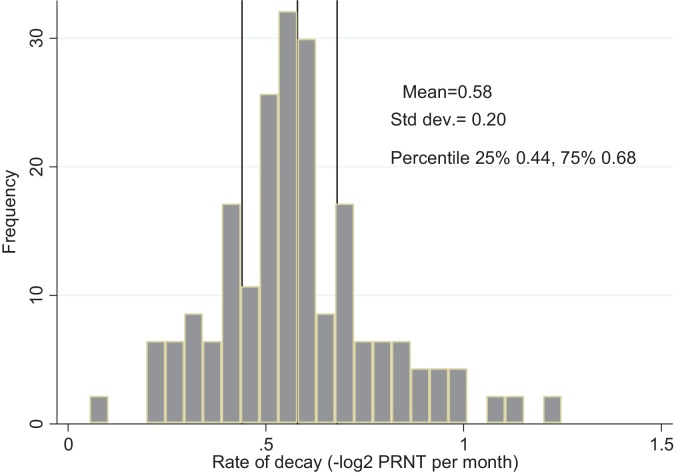
A histogram of the distribution of individual rates of decay showing the overall mean (standard deviation; inter-quartile range), −log_2_PRNT/month.

The distribution of individual rates of decay of RSV PRNT titres (shown in Fig. 3) is unimodal with mean −0.58 (i.e. half life of 36 days), variance 0.04, and IQR −0.44–0.68, i.e. 25% of the sample had a half life of maternal antibodies of ≥48 ≤31 days.

Analysis of covariates on the rate of decay showed no statistically significant effect on underweight <2.5 kg vs normal weight >2.5 kg (log_2_PRNT per month −0.62 vs −0.57; *P* = 0.32) and premature birth vs full term (log_2_PRNT per month −0.66 vs −0.55; *P* = 0.06).

**Fig. 4:**
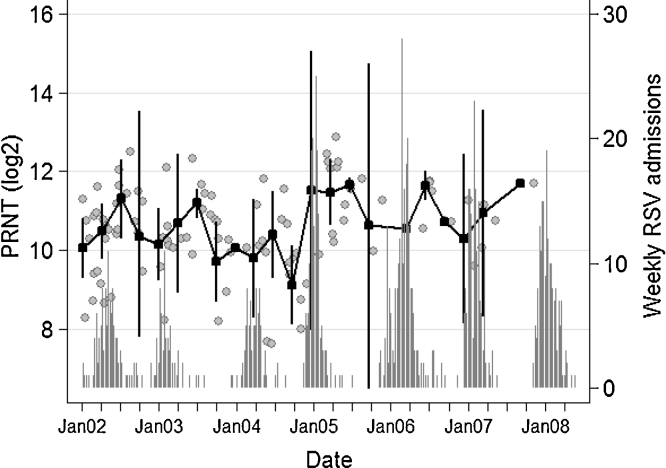
Dynamics of cord titres by time and transmission intensity. Grey symbols denote individual cord titres by date of birth, black square markers the mean cord titre by quarter (95% CI), and the vertical bars show the number of RSV IFAT positive paediatric severe or very severe pneumonia admissions to Kilifi District Hospital 2002–2008.

Further analysis of the cord neutralisation titres by distributing them against date of collection and overlaying with weekly RSV cases admitted to Kilifi District Hospital is shown in Fig. 4. The results suggest that children born at the end and first quarter after an RSV epidemic have higher titres of RSV neutralising antibodies 10.8 log_2_PRNT (SD: 1.06) relative to children born mid inter-epidemic and at the early stage of the epidemic10.0 log_2_PRNT (SD: 1.01) (*P* = 0.043, *t* = −2.09).

## 4 Discussion

RSV is a major cause of severe respiratory disease among infants under 6 months of age [1]. Studies have found that infants born with high levels of RSV specific maternal antibodies are afforded a degree of protection against the development of severe RSV associated pneumonia during the first months of life when the risk of severe disease is greatest [8,17]. Hence, maternal vaccination to boost protective specific antibodies in early infancy represents a plausible disease prevention strategy for RSV. A number of maternal vaccines based on the RSV F protein are now under-development [18] (http://www.clinicaltrials.gov/ct2/show/study/NCT01905215). This study was undertaken to define the baseline characteristics of maternal RSV neutralising antibody transfer and duration in support of the maternal vaccination intervention strategy (http://sites.path.org/vaccinedevelopment/respiratory-syncytial-virus-rsv/vaccine-development/).

In a low income predominantly rural population we estimated a mean concentration of cord neutralising antibody level of 10.6 log_2_PRNT. These results are slightly lower compared to those found in a recent study conducted in Bangladesh [19]. However, other studies reported neutralising antibody titres lower than our estimates [20,21]. We also found that, the cord antibody level is related to birthweight and gestational period; which are some of the factors reported to influence transplacental antibody transfer [22]. This indicates that infants born prior to 28 weeks are less likely to gain antibody levels since RSV specific antibodies do not readily cross the placenta before the 28th week of gestation.

We identified the rate of decay to be −0.58 log_2_PRNT per month in the first 6 months of life, indicating a half life of 36 days. Since the rate of decay might be affected by RSV transmission we compared rates for individuals whose samples were collected within or outside of RSV epidemic periods and found a faster rate of decay (−0.67 log_2_PRNT per month; half-life 31 d) for the latter, though the difference was not statistically significant. However, we also noted that the rate of decay in those under 4 m of age in the total sample (where maternal antibodies are highest and least likely to be influenced by transmission) was relatively high (−0.64 log_2_PRNT/month). Thus, we conclude that the rate of decay of RSV specific maternal neutralising antibodies is between −0.58 and −0.67 log_2_PRNT titres per month, i.e. with average half-life of 36–31 days.

The level of specific RSV neutralising antibody required to prevent disease is not precisely known, but indirect estimates of a protective threshold are reported as 6 and 7.5 log_2_PRNT [10,20]. Our study suggests the vast majority of individuals to be born with titres above this threshold, and furthermore, that over 50% of individuals remain so for around 5–6 m. The indication is that raising levels of cord RSV antibody by maternal vaccination above the median level identified in this study would provide protection to most infants over the first 5–6 m of life deemed to be the most critical for life-threatening RSV disease. Clearly, more work is required to estimate directly the level of protective neutralising antibodies.

Our findings also indicate that the rate of decay of maternally derived neutralising antibodies is independent of starting (cord) level. This result is of significance since it suggests a constant increase in benefit from boosting titres, i.e. for each doubling rise in antibody an increase in duration above a putative protective threshold of around 50 days. This means, a theoretical RSV vaccine which increases maternal antibody titres by approximately 4 fold, would shift the age frequency curve of RSV disease to the right by about 100 days. The above assertion assumes that cord RSV antibody boosted by a maternal vaccine would decay at the same rate as cord antibody of the same level passively transferred from the mother in the absence of vaccination.

Furthermore, although based on a small sample size, we find some evidence for an inverse relationship between population level neutralising antibody titres (inferred from cord sera) and population level virus transmission. RSV specific neutralising antibodies decline in the absence of exposure and increase following an increase in virus transmission at population level. As suggested by Stensballe, this implies that a decline in herd immunity may establish the conditions necessary for the spread of the virus [20].

Our study has a number of limitations. The results are appropriate to a single rural developing country population. Future studies would be of use to provide comparative data in sub-Saharan Africa, in the Indian sub-continent, and in both urban and rural populations. The level and duration of maternal antibody may also vary according to concurrent maternal infections such as HIV and malaria, which are all important factors which this study was not designed to investigate. Measurement error in determining gestational age and hence the pravelence of prematurity could be addressed in future studies through use of standard ultrasound procedures. RSV infects very early in life and the influence of transmission on the estimated rate of decay is problematic. We have taken steps to remove the effect of RSV infection on estimating the rates of decay and determined a realistic range within which the real rate lies. However, there remains the possibility that early infections in the presence of moderate to high titre maternal antibody might not have been detected. Analysis of cord level in relation to prevalent RSV group within a population has not been undertaken. Finally, neutralisation assays are frequently based on RSV A2 virus (without comparison with other strains or alternative group) and not standardised between laboratories and this complicates comparison of results between studies in different locations.

## 5 Conclusions

The results of this study provide precise estimates of the level and duration of maternal RSV neutralisation antibody transfer for a rural developing country population. Similar studies are required in a range of settings. Interpretation of these results requires better understanding of the relationship between the level of RSV-specific neutralising antibody and protection from infection and disease, and the capability of maternal vaccines to boost specific RSV protective antibodies in relation to existing antibody levels.

## Authors’ contributions

JUN designed, implemented the study, performed initial analysis and drafted the manuscript.

CS provided training in performing neutralisation assays, data analysis and reviewed the manuscript.

MM performed neutralisation assays.

PK performed neutralisation assays.

PM conducted data analysis and reviewed the manuscript.

JAS designed the birth cohort study and reviewed the manuscript.

DJN designed the study, conducted data analysis and reviewed the manuscript.

## Conflict of interest statement

DJN has received research support from GlaxoSmithKline. All other author(s) declare that they have no conflict of interest.

## Figures and Tables

**Table 1:** The rate of decay, mean duration and Half-life for different categories of infants in a Kilifi Birth Cohort.

Category	Rate of decay(log_2_PRNT/month)	Mean duration in days	Half-life in days
Overall	−0.58	52	36
Within epidemic	−0.56	54	37
Outside epidemic	−0.67	45	31
<4months	−0.64	47	32
>4months	−0.51	59	41
